# Precision vs. cost: Endoscopic ultrasound-guided vs. conventional pancreatic cyst drainage in a resource-limited setting

**DOI:** 10.1055/a-2821-8219

**Published:** 2026-03-17

**Authors:** Abeer Altaf, Javeria Salman, Muhammad Asim, Muhammad Umair Tahseen, Bushra Ayub, Arif Siddiqui, Shanil Kadir, Mehreen Siyal, Asma Yaseen, Noval Zakaria, Naseer Ahmed, Fahad Kakar, Marwan Elaqaad, Saad Niaz

**Affiliations:** 1 Sindh Institute of Advance Endoscopy and Gastroenterology (SIAG), Dr. Ruth K. M. Pfau Civil Hospital Karachi, Karachi, Pakistan

**Keywords:** Pancreatobiliary (ERCP/PTCD), ERC topics, Endoscopic ultrasonography, Pancreas, Fine-needle aspiration/biopsy, Intervention EUS

## Abstract

**Background and study aims:**

Pancreatic pseudocyst is a frequent complication of pancreatitis. Endoscopic ultrasound (EUS) being the standard approach for drainage, along with conventional transgastric (CTG) remains an alternative, but comparative real-world data from resource-limited settings remain scarce.

**Patients and methods:**

A retrospective review was conducted at a tertiary-care center in Pakistan from 2015 to 2025. Patients undergoing pseudocyst drainage via CTG or EUS were compared for demographic variables, technical success, complications, and procedure costs. Logistic regression was used to identify predictors of either modality for drainage.

**Results:**

A total of 164 patients underwent pseudocyst drainage, including 96 (58.5%) treated with EUS-guided drainage and 68 (41.5%) with CTG. Mean age was 32.76 ± 16.65 years (range 4–77), with 42 pediatric patients. Abdominal pain was the most common presenting symptom. Pseudocysts treated with CTG were significantly larger than those treated with EUS (15.02 ± 8.20 mm vs. 8.49 ± 2.53 mm; *P* < 0.001). Technical success rates were comparable between groups (EUS 96.9% vs. CTG 98.5%; *P* = 0.499), with no significant difference in complications or 6-month clinical outcomes. EUS-guided procedures incurred higher instrument costs (PKR 330,500 vs. PKR 197,500). Larger cyst size was independently associated with CTG selection, whereas EUS was more commonly used in hospitalized patients.

**Conclusions:**

EUS-guided and conventional transgastric drainage demonstrate comparable safety and efficacy. In resource-limited settings, CTG remains a cost-effective option in appropriately selected patients.

## Introduction

Pseudocyst is a frequent complication following pancreatitis episode, with its incidence ranging from 5% to 16% in the setting of acute pancreatitis to increasing to 20% to 40% in cases of chronic pancreatitis 1 . The main pathology behind formation of pseudocyst is disruption of the pancreatic duct (PD), either due to injury or inflammation, causing extravasation of the pancreatic enzymes into the parenchyma, eventually forming collection 2 .

Not every candidate is a case for drainage secondary to spontaneous resolution of the pseudocyst in majority. The American College of Gastroenterology recommends drainage of pseudocyst only if it is symptomatic, namely pain, persistent vomiting, fever, early satiety, and weight loss 3 . Drainage of symptomatic pseudocyst can be done percutaneously, endoscopically, and surgically. Endoscopic drainage of the pancreatic pseudocyst is further divided into two options, depending on cyst relationship with the PD: transmural and transpapillary. For transpapillary, the relationship between the cyst and PD is established with evidence of duct disruption; hence, an endoscopic retrograde cholangiopancreatography-mediated stent is placed to bridge the area and seal the point of leakage. For transmural, there are two further techniques, cystogastrostomy (CTG) and endoscopic ultrasound (EUS)-guided cyst drainage. It is imperative for presence of an external bulge in the gastric or duodenal lumen for adequate drainage of pancreatic pseudocyst via conventional endoscopy, a requirement not needed for EUS-guided cystogastrostomy. In addition, EUS also helps in diagnostic imaging of regional vasculature. Some trials and meta-analyses show better clinical and technical success rates for EUS vs conventional endoscopic technique 4 5 . On the other hand, other studies revealed comparable short- and long-term success rates for both techniques 6 . Although EUS is the standard approach, several studies have compared technical and clinical outcomes of conventional and EUS-guided drainage. However limited data exist on their cost-effectiveness in public-sector, resource-constrained settings. Most available literature focuses on high-resource environments, which may not reflect economic and logistical realities of government-funded institutions in developing countries.

Our aim was to evaluate both transmural pseudocyst drainage techniques, endoscopic and EUS-guided, assess technical success rates, safety, complications of each of the procedures and gauge their cost effectiveness in a government-funded tertiary care setting in Pakistan.

## Patients and methods

### Study design

This was a retrospective observational study done in the Department of Surgical Unit-IV Endoscopy Unit and Sindh Institute of Advance Endoscopy (SIAG) at Dr. Ruth K. M. Pfau, Civil Hospital Karachi, Pakistan. Data were collected over a period of 10 years from January 2015 to January 2025 after receiving institutional ethical review exemption (IRB-3968/DUHS/EXEMPTION/2025/219). All patient data used in this study were de-identified to ensure confidentiality and anonymity, and access to the dataset was restricted to authorized research personnel only.

### Objectives

#### Primary objective

The primary objectives were to assess and compare the technical success rate, defined as successful placement of transmural stents with effective pseudocyst drainage between conventional CTG vs. EUS-guided pseudocyst drainage.

#### Secondary objectives

Secondary objectives were to assess and compare safety and clinical success of both techniques for pseudocyst drainage and to compare cost-effectiveness of both procedures.

### Definitions

Technical success rate was defined as successful access and drainage of the pancreatic pseudocyst with appropriate stent placement confirmed during the procedure.

Clinical success was defined as improvement or resolution of presenting symptoms at 6-month follow-up, without the need for repeat drainage, surgical intervention, or occurrence of delayed complications.

Adverse events (AEs) were defined and categorized according to American Society for Gastrointestinal Endoscopy (ASGE) lexicon for endoscopic AEs 7 . Events were classified as mild, moderate, or severe based on their clinical impact, need for therapeutic intervention, and duration of hospitalization. Mild events included those requiring minimal or no intervention (e.g., self-limited bleeding or transient fever). Moderate events were defined as those necessitating endoscopic or radiologic intervention without surgery or prolonged hospitalization (e.g., stent migration). Severe events (e.g., perforation) were defined as those requiring surgical management, prolonged hospitalization, or resulting in death.

Cost per successful drainage was calculated using total procedure-related cost including all disposable and reusable equipment used during endoscopic drainage procedures, such as the endoscope, stents, balloon dilators, guidewires, and cystotomes, excluding operator charges and fixed hospital overheads.

Procedure-related mortality was defined as any death occurring within 30 days of the endoscopic intervention that could be directly or indirectly related to the procedure or procedure-related complications.

Collections were classified as pancreatic pseudocysts or walled-off necrosis (WON) and were included if they demonstrated a mature wall and were amenable to endoscopic drainage (i.e., minimal solid debris). Fluid appearance and content was documented for all cases.

### Procedures

#### Conventional endoscopic technique

**Fig. 1:**
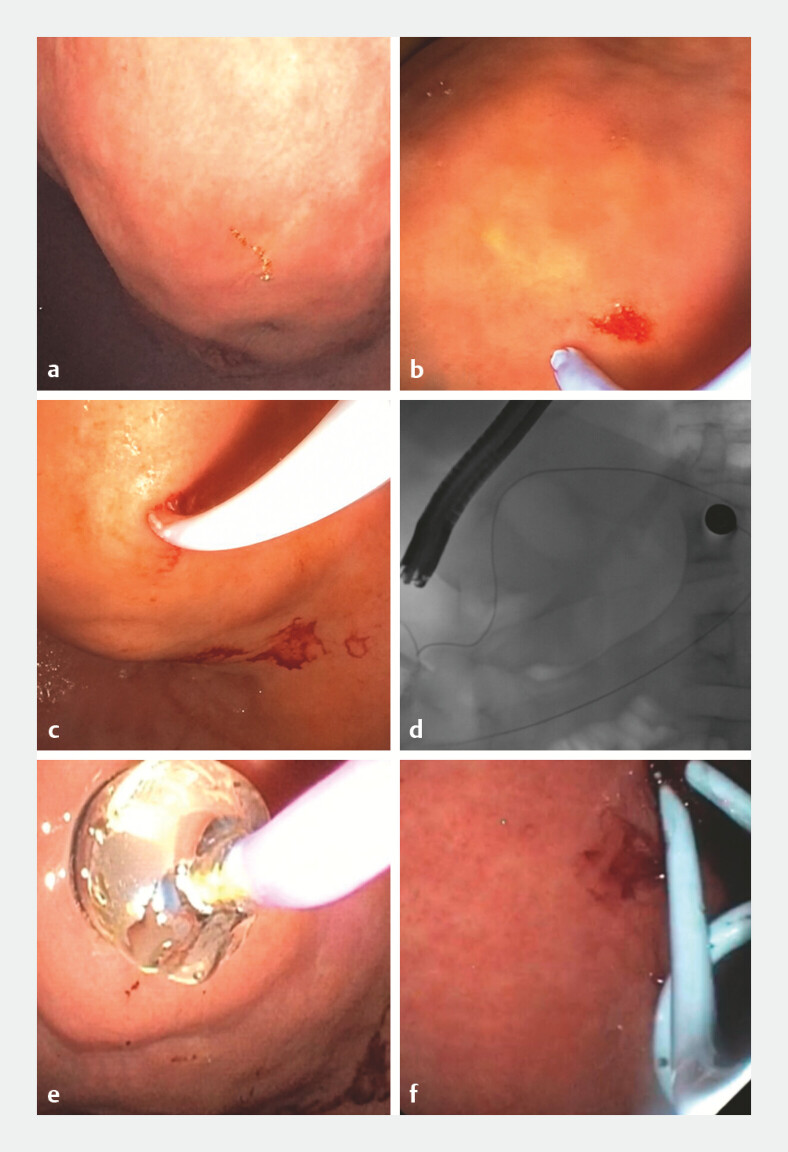
Conventional technique. **a** Bulge identification. **b** Incision using needle knife. **c** guidewire insertion into the cyst. **d** Guidewire coiling as seen on fluoroscopy. **e** Tract dilation using CRE balloon. **f** Double pigtail stent placement under fluoroscopic guidance.

For the endoscopic technique ( Fig. 1 ), a duodenoscope (Olympus TJF therapeutic 190) was used. The duodenoscope is a lateral-viewing instrument, which offers the advantage of a front-on approach to the bulge and availability of an elevator mechanism, facilitating controlled guidewire manipulation and easier, more stable stent placement during conventional transgastric drainage. The duodenoscope is a lateral-viewing instrument, which was the established, standardized scope used for this procedure at our center during the study period. A distinct bulge needed to be seen within the lumen on the stomach or duodenum and then an incision was made using a needle knife (RX Needle Knife XL (Boston Scientific), a 0.035-inch guide guidewire was inserted within the cyst, generous looping was done as confirmed via fluoroscopy, and the site of incision was dilated using CRE balloon dilator (Boston Scientific 8–10 mm). A stent pusher (10F Microtech) was introduced, allowing the second guidewire (0.025 inch) to be placed in the cavity, following which two plastic double pigtail 10F X 5 cm stents (Boston Scientific) were deployed to facilitate drainage of the cyst.

#### EUS-guided pseudocyst drainage

**Fig. 2:**
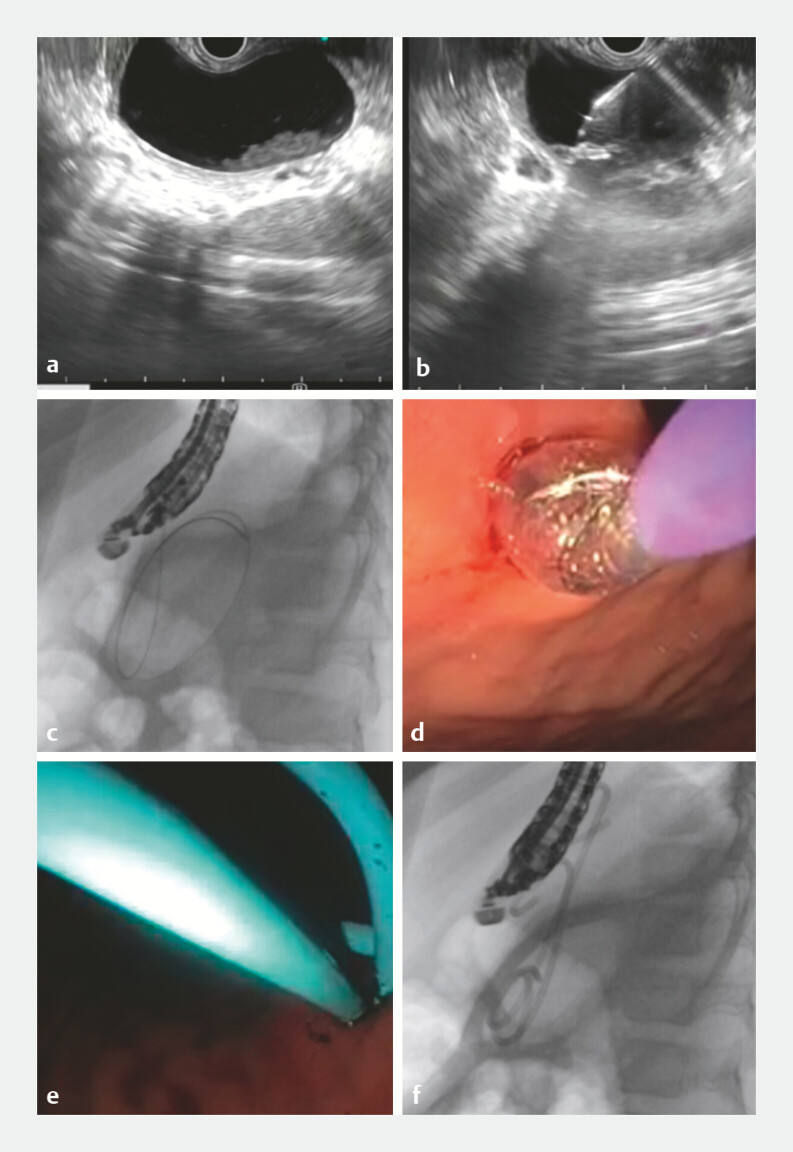
EUS-guided drainage. **a** Cyst identification on EUS. **b** FNA needle puncture. **c** Guidewire insertion and coiling inside the cyst. **d** Fistula tract dilation with CRE balloon. **e** , **f** Stent placement under EUS and fluoroscopic guidance.

All EUS-guided procedures ( Fig. 2 ) were done from the stomach or proximal duodenum using the echoendoscope (Olympus UCT-180 linear scope). Surrounding vasculature was delineated using color Doppler mode. After assessing cyst size and location, a 19-gauge needle (19G fine-needle aspiration needle, Boston Scientific) was introduced to create a fistula between the pseudocyst and the stomach or duodenum. Using both EUS and fluoroscopic guidance, a 0.025-inch guide wire was introduced through the needle and coiled within the pseudocyst, followed by dilation of the fistulous tract initially with a 6F cystotome (Taewong) (The 6F cystotome was systematically included in all EUS cases to ensure controlled, uniform access and tract stability, reflecting the standardized, multistep institutional protocol at our center), followed by a CRE balloon dilator (Boston Scientific 6–8 mm). A 7F stent pusher (Boston Scientific) was introduced and a second guidewire was inserted, and eventually, two plastic double pigtail 7F x 4 cm or 7F x 6 cm stents (Boston Scientific) were placed.

### Data collection method

**Fig. 3:**
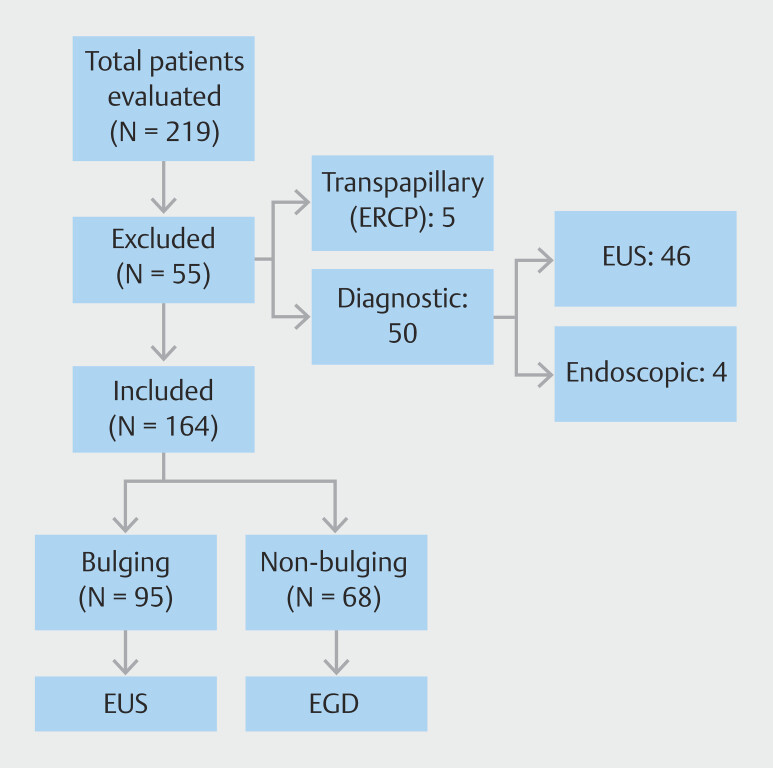
Flow diagram outlining patient selection, inclusion and exclusion criteria, and allocation to EUS-guided or conventional transgastric drainage groups.

We conducted a retrospective review of endoscopic procedure reports performed between January 2015 and January 2025 at our tertiary care center. Using our electronic database, a keyword-based search was performed with the terms "pseudocyst," "bulge," and "cystogastrostomy" to identify relevant patients. All retrieved reports were then manually reviewed to determine relevance for pancreatic pseudocyst drainage. Patients were included if pseudocyst drainage was done using either transmural approach. After applying the inclusion and exclusion criteria, a final cohort of 164 patients was selected for analysis. The detailed patient selection process is summarized in Fig. 3 .

### Study setting

For each patient, the following labs were assessed prior to cyst drainage: complete blood count, renal and liver profiles, and coagulation parameters. Imaging was performed using contrast-enhanced computed tomography (CT) of the abdomen and/or magnetic resonance imaging with magnetic resonance cholangiopancreatography sequences to assess cyst relation with the PD. Patients with large pseudocysts more than 10 cm—conventionally classified as “giant”—were first examined by conventional endoscopy because they are more likely to produce a luminal bulge, making them suitable for drainage via the CTG technique. If the patient had an indication for cyst drainage, but none of the above criteria were met, they then proceeded with EUS-guided drainage. All patients were seen and approved by our anesthesiologist team. The study included patients with symptomatic pseudocysts requiring drainage.

### Exclusion criteria

Patients were excluded if they had: 1) ASA (American Society of Anesthesiologists) class IV or above suggestive of hemodynamic instability; 2) a procedure performed for diagnostic purposes; 3) transpapillary drainage or unencapsulated collection; 3) a collection > 1 cm away from the gastroduodenal tract; 3) collections with pseudoaneurysm; 4) uncorrected coagulopathies; or 5) incomplete data.

### Follow-up and outcomes assessment

As per standard institutional protocol, all patients undergoing pancreatic pseudocyst drainage were scheduled for follow-up evaluations on post-procedure days 1 and 7, at 4 weeks, and at 6 months to assess for symptom resolution, episodes of recurrence, reintervention, and delayed complications using both clinical records for symptom resolution and follow-up abdominal imaging (primarily abdominal ultrasound, supplemented by CT scans when clinically necessary) to confirm complete pseudocyst resolution. Routine follow-up endoscopy was not performed unless clinically indicated (e.g., suspected recurrence, stent migration, or planned stent removal).

For this retrospective study, data from these follow-up visits were obtained from electronic medical records and endoscopy follow-up logs. Patients who reported no symptoms and had no complications were categorized as having achieved clinical success.

### Statistical analysis

Categorical variables such as patient demographics, clinical characteristics, and outcomes, were expressed as frequencies and percentages and were analyzed using the Chi-square test. Continuous variables were presented as means with standard deviations compared using the independent t-test. Multivariate logistic regression analysis was conducted to identify predictors associated with the choice of drainage modality (EUS-guided vs. conventional endoscopic transgastric drainage). *P* < 0.05 was considered statistically significant. All analysis was done on SPSS version 25.0 (IBM Corp., Armonk, New York, United States).

## Results

A total of 164 patients underwent therapeutic pancreatic pseudocyst drainage at our center between 2015 and 2025. Of these, 96 patients (58.5%) had pseudocyst drained via EUS and 68 patients (41.5%) received CTG. Age ranged from 4 to 77 years (mean±SD: 32.76±16.65 years), with 42 patients aged less than 18 years. There was a predominantly male population seen overall (90 males vs 74 females). Abdominal pain was seen as the most common presenting symptom in 127 patients (77.4%), 148 of the patients (90.2%) had pseudocyst secondary to acute pancreatitis, followed by 10 (6.1%) with chronic pancreatitis and six patients (3.7%) developed pseudocyst due to trauma. Mean largest cyst dimension was smaller in the EUS group compared with the CTG group (8.49±2.53 mm vs. 15.02±8.20 mm). On fluid analysis, the majority of aspirates were clear (57.8%) or straw-colored (32.0%), consistent with uncomplicated pseudocysts. A smaller subset demonstrated purulent (9.5%) or blood-stained (0.7%) fluid, suggesting presence of infected or hemorrhagic collections in a minority of cases. On EUS, internal septations were observed in only 2.5% of collections, indicating that most had a single cavity with homogenous content. These findings collectively suggest that although the majority of cases represented simple pseudocysts, a small proportion contained features compatible with WON. This suggests that patients selected for EUS-guided drainage had comparatively smaller cysts than those undergoing conventional transgastric drainage, highlighting a potential difference in case selection or anatomical suitability between the two approaches. A detailed overview comparing the two groups (EUS vs CTG) is shown in Table 1 .

**Table:** **Table 1** Baseline demographic and clinical characteristics of patients undergoing pancreatic pseudocyst drainage, comparing EUS-guided and conventional transgastric (CTG) approaches.

Baseline characteristics	EUS (N = 96)	CTG (N = 68)	*P* value
Age mean (years)	35.65±16.89	28.69±15.53	0.254
Number of patients aged < 18 years	19 (19.8%)	23 (33.8%)	0.043
Gender (M/F)	52/44 (54.2%/45.8%)	38/30 (55.9%/44.1%)	0.828
Cause of pancreatitis			
Biliary	74 (77.1%)	52 (76.5%)	0.449
Alcoholic	8 (8.3%)	6 (8.8%)	
Autoimmune	1 (1.0%)	1 (1.5%)	
Idiopathic	4 (4.2%)	2 (2.9%)	
Largest cyst dimension mean (cm)	8.49±2.53	15.02±8.20	< 0.001
Vomiting	48 (50%)	40 (58.8%)	0.264
Abdominal pain	68 (70.8%)	59 (86.8%)	0.016
Recurrent pancreatitis	19 (19.8%)	8 (11.8%)	0.172
Cause of pseudocyst formation*			0.449 *
Acute pancreatitis	85 (88.5%)	63 (92.6%)	
Chronic pancreatitis	6 (6.3%)	4 (5.9%)	
Post-traumatic	5 (6.3%)	1 (1.5%)	
* *P* value corresponds to the overall comparison of the distribution of etiologies (acute, chronic, and post-traumatic pancreatitis) between the EUS and CTG groups using the Chi-square test. Categorical variables are presented as number and percentage (N [%]) and were compared using the Chi-square or Fisher’s exact test, as appropriate. Continuous variables are expressed as mean±standard deviation and compared using the independent t-test. *P* < 0.05 was considered statistically significant. CTG, cystogastrostomy; EUS, endoscopic ultrasound.			

Technical success was achieved in 93 patients (96.9%) in the EUS-guided group vs 67 (98.5%) in the CTG group ( *P* = 0.499), suggesting no statistical difference in successful drainage using either procedure. Logistic regression analysis was performed to identify factors associated with choice of pseudocyst drainage (EUS vs CTG). Cyst size was significantly associated with procedure type, with larger cysts more likely to be treated using CTG (odds ratio [OR]1.30; 95% confidence interval [CI] 1.18–1.43; *P* < 0.001). This indicates a strong association between increasing cyst size and choice of CTG over EUS. Post-procedure hospitalization was significantly associated with increased odds of receiving EUS-guided drainage (OR 3.17; 95% CI 1.28–7.89; *P* = 0.013), suggesting that EUS may be preferentially utilized in more complex or high-risk cases requiring closer post-procedure observation or inpatient care. Although not statistically significant, there was a trend toward CTG-guided drainage in patients presenting with abdominal pain (OR 0.47; 95% CI 0.20–1.10; *P* = 0.083) and those younger than 18 years of age (OR 0.52; 95% CI 0.25–1.11; *P* = 0.075), indicating potential clinician preference in these subgroups. AEs were infrequent and comparable between both groups. Minor self-limited bleeding occurred in four patients (2.4%)—two in each group—and resolved without need for intervention. Stent migration was observed in two patients (1.2%), one from each group, and was classified as a moderate AE per ASGE criteria; both were managed endoscopically without sequelae. No perforation or procedure-related mortality was reported. Overall, the majority of AEs were mild, with no severe complications observed.

A detailed comparison of clinical outcomes between the two groups is presented in Table 2 .

**Table:** **Table 2** *Clinical outcomes observed in patients undergoing endoscopic ultrasound-guided versus conventional transgastric (cystogastrostomy) drainage procedures.*

Outcome measure	EUS N (%)	CTG N (%)	*P* value
Technical success rate			0.499
Yes	93 (96.9%)	67 (98.5%)	
No	3 (3.1%)	1 (1.5%)	
Procedure failure			0.499
Yes	3 (3.1%)	1 (1.5%)	
No	93 (96.9%)	67 (98.5%)	
Post procedure bleeding (minor)			0.726
Present	2 (2.1%)	2 (2.9%)	
Absent	94 (97.9%)	66 (97.1%)	
Significant bleeding			–
Present	0 (0%)	0 (0%)	
Absent	96 (100%)	68 (100%)	
Stent migration			0.805
Yes	1 (1%)	1 (1.5%)	
No	95 (99%)	67 (98.5%)	
Post-procedure perforation			–
Present	0 (0%)	0 (0%)	
Absent	96 (100%)	68 (100%)	
Post-procedure hospitalization			0.018
Yes	24 (25%)	7 (10.3%)	
No	72 (75%)	61 (89.7%)	
Procedure-related 30-day mortality			–
Present	0 (0%)	0 (0%)	
Absent	96 (100%)	68 (100%)	
Data are presented as number and percentage (N [%]). Statistical comparisons were performed using the Pearson chi-square test. *P* < 0.05 was considered statistically significant. CTG, cystogastrostomy; EUS, endoscopic ultrasound.			

One patient from the CTG group underwent cyst puncture but developed bleeding, which resolved spontaneously; however, to ensure procedure safety, the technique was converted to EUS to visualize surrounding vessels on Doppler imaging before safely draining the cyst. Four patients who initially were referred for EUS pseudocyst drainage were switched to the CTG group due to presence of a large intragastric bulge, making direct endoscopic access feasible without the need for EUS guidance.

Of the failures, one patient belonged to the CTG group where after puncture no adequate fluid was seen draining, and in three patients who failed in the EUS group, one had significant gastric edema that limited access and two had device-related technical failure (cystotome and stent pusher), with one patient in the former group referred for a new CT scan and followed up in clinic; all three patients in the latter group underwent EUS-guided drainage on a later day.

Among patients successfully followed at 6 months, clinical success was reported in 69 of 95 patients (72.2%) in the EUS group and 47 of 68 patients (69.1%) in the endoscopic group. One patient (1.1%) in the EUS group reported recurrence; however, repeat ultrasound revealed spontaneous resolution, hence no reintervention was required. There were no delayed complications reported in either group during the follow-up period. A considerable number of patients from both groups were lost to follow-up (26.3% EUS vs. 30.8% CTG), limiting long-term outcome assessment. The apparent decline reflects loss to follow-up rather than true recurrence or failure. Our institution is a tertiary referral center, receiving patients from multiple secondary and district-level hospitals across the region. After successful drainage and clinical stabilization, many patients are referred back to their primary hospitals for continued follow-up, which partly explains loss to long-term follow-up observed in this study.

**Fig. 4:**
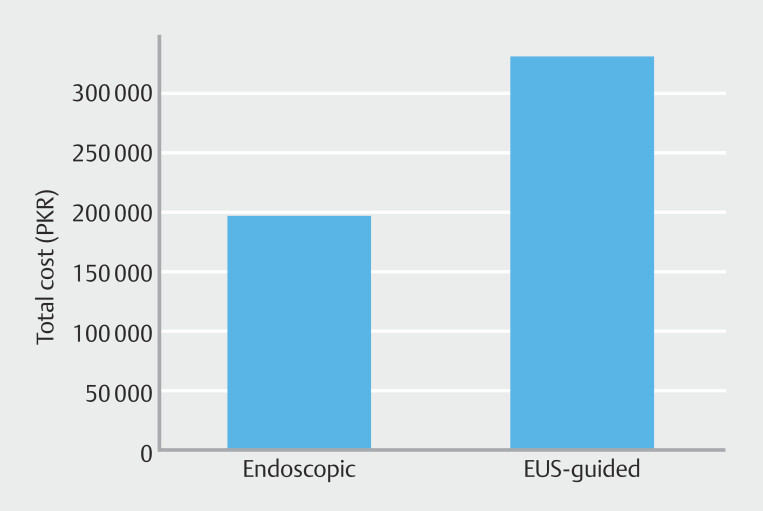
Comparison of total instrument-related procedural costs between conventional transgastric (CTG) and EUS-guided pseudocyst drainage techniques, expressed in Pakistani Rupees (PKR) based on 2025 pricing.

For cost comparative analysis, only the cost of procedural gadgets was considered. The average device-related cost per procedure was approximately USD 700 (PKR 197,500) for the CTG group and USD 1,170 (PKR 330,000) for the EUS-guided group, currency conversion based on average exchange rate of 1 USD = 282 PKR at time of analysis. A comparative overview is presented in Fig. 4 . The higher cost in the EUS group was primarily due to use of specialized accessories such as the FNA needle and cystotome. A detailed breakdown of each itemized accessory is shown in Supplementary Table 1.

## Discussion

Our study reviews outcomes of pancreatic pseudocyst drainage performed over a decade (2015–2025) at our tertiary care center comparing the two modalities: CTG and EUS.

Both groups demonstrated high technical success rates, with a single failure observed in the CTG group (98.5%) and three in the EUS group (96.8%). Although numerically lower, this difference was not statistically significant. A meta-analysis conducted by Pamonata et.al also demonstrated comparable efficacy between conventional endoscopic and EUS-guided approaches in terms of short- and long-term successes 6 .

The groups had no observed difference in mortality, and complication rates were similar, including minor bleeding and stent migration. No major AEs were recorded in immediate or 6-month follow-up. Our findings partially align with previous studies, such as those summarized by Teoh et al., which reported similar clinical success and AE rates between EUS-guided and CTG techniques in selected patient groups, although technical success was often higher with EUS 7 . Furthermore, Park et al. have demonstrated that EUS guidance provides technical advantages in patients without clear luminal bulge or with intervening vasculature, supporting the need for careful patient selection 8 .

The high success rate for conventional drainage in our cohort further promotes that in cases in which the pseudocyst exhibits a visible luminal bulge with favorable anatomical access, conventional drainage remains an appropriate and safe technique, with technical and clinical outcomes comparable to EUS-guided drainage in carefully selected patients. Although conventional drainage can suffice for bulging, anatomically favorable lesions, we acknowledge that performing EUS-guided drainage provides distinct advantages beyond just access, including pre-procedure Doppler evaluation to rule out intervening vasculature for safety, and visualization of the main PD and surrounding pancreatic parenchyma, which is crucial for full disease assessment and excluding malignancy or ductal disconnection. The slightly higher failure rate and more post-procedure hospitalizations in the EUS group likely reflect the fact that EUS was more frequently used in complex, non-bulging, or high-risk pseudocysts where conventional access was limited or unsafe 9 . Logistic regression analysis revealed that cyst size and post-procedure hospitalization were significantly associated with choice of drainage modality. The finding that cyst size was significantly associated with CTG selection and EUS was more frequently chosen in hospitalized patients largely mirrors the institutional selection algorithm (favoring CTG for large, bulging cysts and EUS for non-bulging or complex ones), rather than indicating independent causal factors or superiority of technique. We emphasize that the two groups, therefore, represent inherently different patient populations and our selection bias is consistent with prior studies.

A small subset of patients underwent crossover between techniques based on intra-procedural findings. Four cases initially planned for EUS-guided drainage were converted to conventional endoscopic drainage due to a prominent luminal bulge measuring > 10 cm, allowing safe direct access, whereas one CTG case was switched to EUS for vascular safety. These crossovers highlight the complementary nature of both methods and underscore the importance of individualized, anatomy-driven decision-making in real-world practice.

This study was conducted in a government-funded tertiary care referral center in Pakistan, a country with a developing economy, yet one where funding in the healthcare system faces the challenge of meeting rising demands within constrained resources. With a GDP per capita of approximately $1,600 10 and public health expenditure accounting for less than 3% of GDP 11 , cost-conscious interventions are critical for ensuring continuous and sustainable access to medical care within the public healthcare institutions.

To assess the financial impact of endoscopic versus EUS-guided pseudocyst drainage, we did a cost evaluation considering only direct procedure costs related to instrument use. Average cost of consumables for conventional endoscopic drainage, as per procurement cost of 2025, was USD 700 (197,000 PKR), whereas EUS-guided drainage averaged USD 1,170 (330,500 PKR) per procedure. Cost analysis in this study was confined to the direct procedure expenses of the index intervention, specifically instruments and accessories used during drainage. Broader cost factors such as anesthesia, total hospital stay duration, costs associated with management of complications, and required follow-up imaging were not included due to the retrospective nature of data collection and lack of standardized, itemized billing for these services in our public-sector institutional records. Although this restricted approach allows for a standardized and reproducible comparison of the immediate procedure cost difference between EUS-guided and conventional drainage, we acknowledge that it may underestimate the overall economic burden and is a limitation of this study. This represents a substantial 45.8% cost difference favoring the conventional approach. Importantly, this cost difference also did not correlate with superior procedure outcome i.e., technical success rates were high and comparable between both groups, with similar complication profiles and no difference in procedure-related mortality. Although this provides a snapshot of comparative procedure expenditure, it may not accurately reflect true historical costs due to considerable financial variations and inflation in Pakistan from 2015 to 2025 12 .

In a public-sector resource-limited environment, this cost gap has significant implications. These findings suggest that in anatomically favorable cases, conventional drainage offers an equally safe and more cost-effective solution, reducing procedure costs by approximately USD 470 (132,00 PKR) per case. Our findings support that in appropriately selected patients—particularly those with visible intraluminal bulging—CTG-guided drainage may be safely and effectively performed at secondary-level centers lacking EUS capabilities. This may not only reduce procedure-related costs but also travel costs, thereby optimizing care delivery closer to the patient's home. If implemented across institutions nationwide, this could result in sizeable savings for the already overly burdened and underfinanced national healthcare system.

EUS drainage in our study was performed exclusively using plastic stents. It is important to note the emergence of lumen-apposing metal stents (LAMSs) in high-resource settings, offering potential benefits such as wider tract creation and reduced need for reintervention in complex collections. However, LAMS technology remains unavailable in Pakistan due to prohibitive cost, which is approximately PKR 1 million (≈ USD 3,500) per unit, making them impractical for routine use in public-sector hospitals in Pakistan. Consequently, plastic stents remain the standard and most cost-effective method for liquid-dominant pseudocysts and simple WON in our resource-limited environment. Our findings, therefore, provide crucial data on the efficacy and safety of plastic stent drainage in a setting where LAMSs are not a feasible option and solid-dominant collections are managed differently.

There are several strengths of this study, including a 10-year large dataset of 164 patients reflecting long-term institutional practice patterns. It not only compares technical success of both techniques but also clinical outcomes and cost estimation in both adult and pediatric cases, which is highly relevant for public health care systems in low- and middle-income countries (LMICs), a context that is underrepresented in the literature. This retrospective review included both adult and pediatric patients; pediatric cases formed 25% of the cohort and were managed using the same institutional protocols, which adds relevance for public health care systems in LMICs. With low complication rates and absence of procedure-related mortality across the board, our study emphasizes safety and feasibility of both approaches in experienced hands. However, there are a few limitations as well. The retrospective nature of the study introduces the likelihood of selection bias and limits control over possible confounders. Being a single-center study reduced generalizability of the results. The relatively small sample size over a long study duration (164 procedures over 10 years, averaging approximately 16 procedures per year, divided between the two techniques) represents a key limitation of this analysis. The modest annual case volume reflects the rarity of pancreatic pseudocyst drainage procedures and referral variability in our setting. This limitation may reduce statistical power to detect subtle differences between groups; however, the extended 10-year inclusion period ensures a representative real-world experience from a tertiary care center in a resource-limited environment. Patients undergoing EUS-guided drainage may have had more complex anatomy, which could have influenced both outcomes and procedure-related costs over possible confounders. In addition, the analysis was restricted to device-related cost of the index procedure; we did not incorporate all associated downstream costs, such as hospital stay or follow-up management, due to inherent data limitations of retrospective review in our public health system. Furthermore, long-term follow-up data showed substantial loss to follow-up patients in both cohorts, making judgment about long-term clinical outcomes difficult. Future prospective studies should evaluate both clinical and economic outcomes. This includes assessment of technical success, clinical success, safety alongside economic evaluations, incorporating both direct medical costs (such as accessories, imaging, hospital stay, and medications) and indirect costs (including patient travel and time off work), with outcomes calculated in terms of incremental cost-effectiveness ratio.

## Conclusions

Although EUS-guided drainage is considered standard of care because of its high precision and adaptability, its routine use in all pseudocyst cases in LMICs such as Pakistan may not be economically practical. Our data suggest that in cases with a clear luminal bulge and accessible anatomy on prior CT imagining, conventional drainage is an appropriate and safe technique, with outcomes comparable to EUS-guided drainage in carefully selected patients. However, EUS remains advantageous for non-bulging, complex, or vascularly adjacent collections. Perhaps a cost-conscious approach involving use of conventional endoscopy for pseudocyst drainage where anatomically feasible is crucial to resource optimization for everyday clinical practice. This study represents one of the largest real-world datasets comparing outcomes for efficacy, safety, and economic implications between both EUS and endoscopic modalities for pancreatic pseudocyst drainage.

## Notes

Correction **Precision vs. cost: Endoscopic ultrasound-guided vs. conventional pancreatic cyst drainage in a resource-limited setting** Abeer Altaf, Javeria Salman, Muhammad Asim et al. Endoscopy International Open 2026; 14: a28218219. DOI: 10.1055/a-2821-8219 In the above-mentioned article table 1 was corrected. This was corrected in the online version on 24.03.2026.

## Supplementary Material

Supplementary Material Zusatzmaterial
