# Correction: Precision vs. cost: Endoscopic ultrasound-guided vs. conventional pancreatic cyst drainage in a resource-limited setting

**DOI:** 10.1055/a-2839-1620

**Published:** 2026-03-25

**Authors:** Abeer Altaf, Javeria Salman, Muhammad Asim, Muhammad Umair Tahseen, Bushra Ayub, Arif Siddiqui, Shanil Kadir, Mehreen Siyal, Asma Yaseen, Noval Zakaria, Naseer Ahmed, Fahad Kakar, Marwan Elaqaad, Saad Niaz

**Affiliations:** 1 Sindh Institute of Advance Endoscopy and Gastroenterology (SIAG), Dr. Ruth K. M. Pfau Civil Hospital Karachi, Karachi, Pakistan



In the above-mentioned article table 1 was corrected. This was corrected in the online version on 24.03.2026.

