# Supplementary material for: Precision vs. cost: Endoscopic ultrasound-guided vs. conventional pancreatic cyst drainage in a resource-limited setting
Source: Endosc Int Open. 2026 Mar 17;14:a28218219. doi: 10.1055/a-2821-8219 (PMC13062678; doi:10.1055/a-2821-8219)
Supplement: Supplementary file 1 — Supplementary Material [file 10-1055-a-2821-8219_28267689.pdf]

Supplementary Table 1 Itemized instrument cost for EUS-guided and CTG Pseudocyst drainage procedures as per procurement cost of 2025.

Procedure	Item	Cost (USD)	Cost (PKR)
**EUS**	19G FNA Needle (Boston Scientific)	317	89,500
	CRE Dilator (Boston Scientific 6–8 mm)	186.2	52,500
	2× Guidewires (0.025)	202.1	57,000
	2× DP 7F × 4/6 cm stents (Boston Scientific)	138.3	39,000
	Stent Pusher 7F (Boston Scientific)	62.1	17,500
	Cystotome 6F (Taewong)	265.9	75,000
	**Total**	**1,171.90**	**330,500**
**Endoscopic**	Needle Knife (RX Needle Knife XL, Boston Scientific)	150.7	42,500
	Guidewires ×2 (0.035 and 0.025)	177.3	50,000
	CRE Dilator (Boston Scientific 6–8 mm)	186.2	52,500
	2× DP 10 Fr × 5 cm stents (Boston Scientific)	124.1	35,000
	Stent Pusher 10F (Microtech)	62.1	17,500
	**Total**	**700.4**	**197,500**

All costs presented in Pakistani Rupees (PKR) and US Dollar (USD) using current conversion of 1 PKR=USD 282.
CTG, cystogastrostomy; EUS, endoscopic ultrasound.
